# Use of external fixation for perilunate dislocations and fracture dislocations

**DOI:** 10.1007/s11751-014-0201-3

**Published:** 2014-10-10

**Authors:** Olga D. Savvidou, Michael Beltsios, Vasileios I. Sakellariou, Andreas F. Mavrogenis, Michael Christodoulou, Panayiotis J. Papagelopoulos

**Affiliations:** 1 Department of Orthopaedics, Athens University Medical School, General University Hospital ‘ATTIKON’, 1 Rimini Street, 12462 Haidari, Greece; 2 Department of Orthopaedics, Thriassio General Hospital, Magoula, Greece

**Keywords:** Carpal, Dislocation, Minimal, External fixation, Ligamentotaxis

## Abstract

The purpose of this study was to review clinical and radiographic outcomes of perilunate dislocations and fracture dislocations treated with external fixation and K-wire fixation. Twenty patients (18 males and two females) with a mean age of 38 years (range 18–59) who had an acute dorsal perilunate dislocation or fracture dislocation were treated with the use of wrist external fixator and K-wires. The injuries included 12 perilunate dislocations, seven trans-scaphoid perilunate fracture dislocations, and one trans-styloid perilunate fracture dislocation. The median time from trauma to operation was 8 h (2–12 h). Indirect reduction via ligamentotaxis was achieved in all perilunate dislocation, and provisional K-wire fixation was added. In five of seven trans-scaphoid perilunate fracture dislocations, indirect reduction was achieved; whereas in the other two as well as in the case of trans-styloid perilunate fracture dislocation, open reduction was required. External fixator was supplemented with K-wires for stabilization of the fractures and the intercarpal intervals. The interosseous and capsular ligaments were not repaired, even after open reduction of fracture dislocations. The mean follow-up was 39 months (range 18–68 months). Range of motion and grip strength were measured. Cooney’s scoring system was used for the assessment of clinical function. Radiographic evaluation included time to scaphoid union, measurement of radiographic parameters (scapholunate gap, scapholunate angle, lunotriquetral gap, and carpal height ratio) and any development of arthritis. The flexion-extension motion arc and grip strength of the injured wrist averaged 80 and 88%, respectively, of the corresponding values for the contralateral wrists. According to Cooney’s clinical scoring system, overall functional outcomes were rated as excellent in four patients, good in eight, fair in six, and poor in two. Eighteen patients returned to their former occupations. Two patients with a trans-scaphoid perilunate injury developed nonunion of the scaphoid; one of them required scaphoid excision and midcarpal fusion. Two patients had radiographic evidence of arthritis. The use of external fixation and provisional K-wire fixation for the treatment of acute perilunate dislocations is associated with satisfactory midterm functional and radiographic outcomes. This minimally invasive treatment option is simple, reliable, and minimally invasive method that provides proper restoration and stable fixation of carpal alignment.

## Introduction

Perilunate dislocations and fracture dislocations are unusual high-energy injuries, which tend to occur in young people and may lead to considerable long-term morbidity due to the development of carpal instability and radiocarpal and midcarpal arthritis. They are often misdiagnosed as simple wrist sprains, and up to 25 % of them are missed during initial presentation at the emergencies [1, 2].

The goal of treatment is reduction of dislocation, internal fixation of the fractures, and ligamentous repair [3–8]. However, open reduction introduce further surgical trauma to capsular and ligamentous structures and is associated with high rate of complications, such as joint stiffness due to capsular fibrosis and delay or failure of proper bone healing because of damage to the blood supply [9, 10]. On the other hand, closed reduction and stabilization with percutaneous pinning only has been associated with high incidence of recurrent instability, carpal incongruity, and development of late post-traumatic arthritis [11].

The purpose of our study was to evaluate the clinical and radiographic outcomes related to the use of wrist external fixator supplemented with K-wires for the treatment of perilunate dislocations and fracture dislocations of the wrist.

## Patients and methods

We performed a retrospective study in a cohort of 20 patients (18 males and two females) presented with perilunate dislocation or fracture dislocation of their wrist between April 2002 and November 2008 that were managed operatively with the use of a wrist external fixator (Pennig Dynamic Wrist Fixator, Orthofix, Italy, and DFS Distal Radius Fixator, Biomet, NJ USA) and Kirschner wires. The mean age of these patients was 38 years (range 18–59). The median time from injury to operation was 8 h (2–12 h). There were 16 right and four left wrists, with the dominant hand involved in 16 cases. The etiology of wrist injury was motor vehicle accident in 10 cases and fall from height in the other 10 cases.

There were 12 dorsal perilunate dislocations, seven trans-scaphoid perilunate fracture dislocations, and one trans-styloid perilunate fracture dislocation. Two fracture dislocations were open, Gustillo II. Nine patients had concomitant injuries that included: olecranon and radial head fracture in one patient, elbow dislocation in five patients, and clavicle fracture in one patient. Three patients suffered from multiple skeletal injuries. Acute post-traumatic median nerve dysfunction was clinically evident in four patients.

**Fig. 1:**
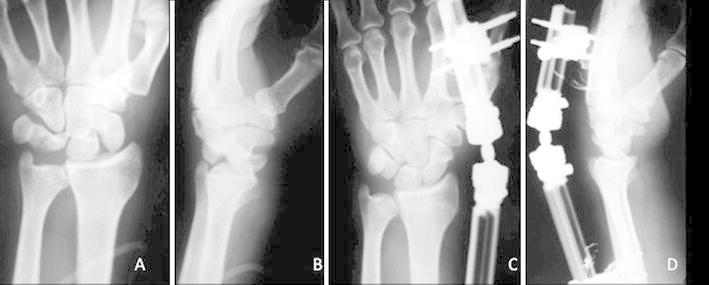
Posteroanterior **(a)** and lateral **(b)** radiographic views of the left wrist of a 59-years-old male with a perilunate dislocation. Posteroanterior **(c)** and lateral **(d)** lateral views of the wrist joint before the application of K-wires, showing successful restoration of normal carpal alignment, which was achieved via ligamentotaxis using a wrist external fixation

The surgery was performed with the patient under brachial plexus block in eight cases and general anesthesia in 12 cases. In all acute perilunate dislocations, the reduction was achieved via ligamentotaxis by applying longitudinal traction using the wrist external fixator (Figure 1a–d).

**Fig. 2:**
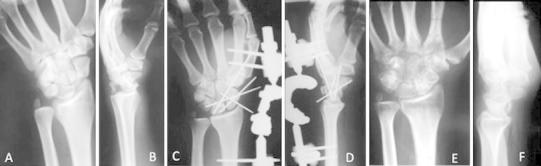
Posteroanterior **(a)** and lateral **(b)** radiographs of a 19-years-old male showing a trans-scaphoid trans-triquetral perilunate fracture- dislocation of the left wrist joint. Postoperative posteroanterior **(c)** and lateral **(d)** projections of the left wrist after reduction of the fracture dislocation and stabilization using an external fixator augmented with percutaneous K-wires. The scaphoid and the triquetral fractures were fixed with K-wires. **e** Posteroanterior and **f** lateral views of the same wrist 10 months postoperatively showing preservation of normal carpal alignment

The external fixator was applied using a standard surgical technique; a stab incision in the frontal plane over the flare of the tubercle of the second metacarpal was made. Soft tissues were separated down to bone and the center of bone with a trocar inserted into the short screw guide in the template (with a handle). The trocar was removed, and a drill guide into the screw guide was inserted. We drilled both cortices with a 2.7-mm drill bit. We inserted the first screw into the second metacarpal in the frontal plane to a depth of about 10 mm. We repeated the above procedure for the distal metacarpal screw. The two radial screws were inserted in the frontal plane through a single 25 mm incision, after blunt dissection down to the bone to avoid injury to the superficial branch of the radial nerve. We applied gentle, gradually increasing manual traction to reduce dislocation and fracture dislocation under image intensification. The double ball-joint was locked, after we confirmed that the distal ball-joint was aligned with the capitate-lunate axis. When the anatomic reduction was achieved, additional K-wires were inserted under fluoroscopic guidance in order to transfix the scapholunate and lunotriquetral intervals (Fig. 2a–g).

**Fig. 3:**
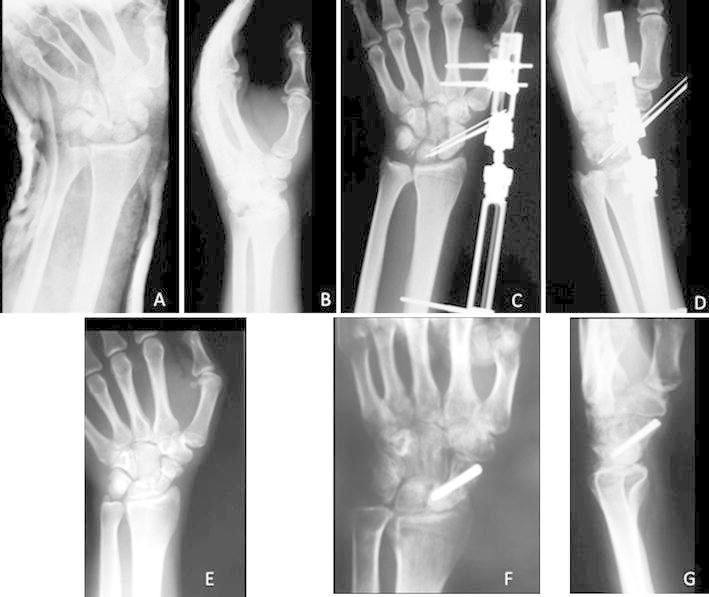
Posteroanterior **(a)** and lateral **(b)** views of the left wrist of a 35-years-old male with a trans-scaphoid perilunate fracture dislocation. Posteroanterior **(c)** and lateral **(d)** radiographs after open reduction through a dorsal approach, application of a wrist external fixator and K-wires. The scaphoid was fixed with K-wires. **e** Three months postoperatively, a scaphoid nonunion was radiographically evident. A cannulated screw was used for the scaphoid nonunion. Posteroanterior **(f)** and lateral **(g)** radiographs of the wrist 10 months postoperatively showed the development of post-traumatic

Closed indirect reduction via ligamentotaxis using wrist external fixator was achieved in five out of seven perilunate fracture dislocations. In two cases, open reduction through a dorsal approach was necessary (Fig. 3a–f). In five out of the seven trans-scaphoid perilunate dislocations, indirect reduction was feasible. Percutaneous fixation of scaphoid fracture with K-wires was then attempted; under fluoroscopic control, the wrist was gently flexed while the fracture was fixed with K-wires, which were advanced from dorsal to volar along the central axis of the scaphoid. The scapholunate and lunotriquetral joints were also stabilized with additional K-wire fixation under arthroscopic assistance. In two out of seven trans-scaphoid fracture dislocations, open reduction through a dorsal approach was necessary. In the case of trans-styloid perilunate fracture dislocation, open reduction was achieved through a volar approach. K-wires were used for fixation of scaphoid fracture and radial styloid fractures, as well as, for stabilization of scapholunate and lunotriquetral intervals. The interosseous and capsular ligaments were not sutured, even after open reduction of the dislocations.

**Fig. 4:**
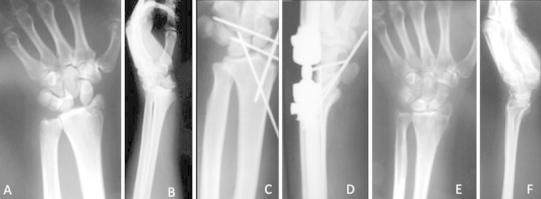
Posteroanterior **(a)** and lateral **(b)** radiographs of a 33-years-old female showing a trans-styloid perilunate fracture dislocation. Postoperative posteroanterior **(c)** and lateral **(d)** views of the same wrist after open reduction and application of external fixator and additional use of K-wires. Posteroanterior **(e)** and lateral **(f)** radiographs 12 months postoperatively showing normal carpal alignment with no signs of arthritis

**Fig. 5:**
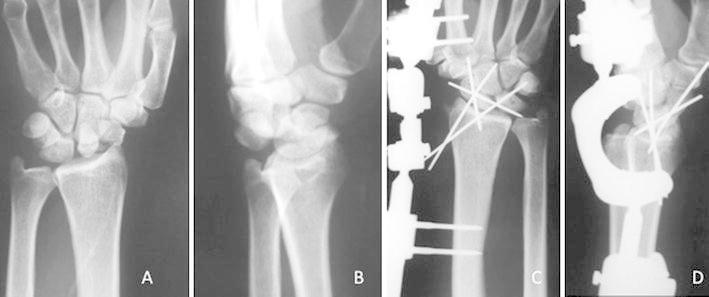
Posteroanterior **(a)** and lateral **(b)** radiographic views of the wrist of a 45-years-old woman showing a trans-scaphoid perilunate fracture dislocation. Postoperative posteroanterior **(c)** and lateral **(d)** radiographs of the wrist after open reduction, external fixation and application of K-wire, demonstrating restoration of normal carpal alignment

 Active-assisted wrist motion was usually allowed at 6 weeks for perilunate dislocations and at 8 weeks for perilunate trans-scaphoid fracture dislocations and for trans-styloid perilunate fracture dislocation. The K-wires transfixing the scapholunate and lunotriquetral intervals were removed at 10 weeks to prevent late lunate subluxation and allow intercarpal ligamentous healing. In patients with a scaphoid fracture that had been fixed with K-wires, the K-wires were left in place until there was radiographic evidence of union. Intensive physiotherapy was started after the scaphoid united, and K-wires had been removed (Figs. 4, 5).

The average follow-up was 39 months (range 18–68 months). Range of motion (flexion and extension, pronation and supination, and radial and ulnar deviation), and grip strength were evaluated at the latest follow-up. Range of motion was measured using a handheld goniometer, and grip strength was measured with a hydraulic dynamometer (JAMA; Asimov Engineering, Los Angeles, CA). Functional evaluation was performed according to the clinical scoring system of Cooney, which consists of four elements (pain, range of motion, grip strength, and function) (Table 1). Points were accumulated for the 4 categories, and the final rating of this system was obtained as follows: excellent, 90–100 points; good, 80–90 points; fair, 65–80 points; and poor, <65 points [12].

**Table 1:** Table describing the Cooney’s clinical scoring chart that was used for the clinical assessment of our patients

Cooney’s clinical scoring chart
*Pain (25 points)*
25: None
20: Mild occasional (climatic or with heavy use)
10: Moderate (with normal use, not at rest)
0: Severe, constant
*Range of motion (25 points): F+E: (degrees)*
25: 140
20: 100–140
15: 70–99
10: 40–69
0: 40
*Grip strength (25 points)*
25: Normal^a^
15: Diminished but 50 % of normal
0: <50 % of normal
*Activities (25 points)*
25: Same activities
15: Restricted activities caused by injured wrist
0: Change of work or sports caused by injured wrist

Excellent 100–90 points; Good 90–80 points; Fair 80–65 points; and Poor <65 points

^a^Normal is contralateral side (minus or plus 10 % depending on dominance) if not disabled. If disabled, then reference is made to normal estimated values with respect to age

Radiographic assessment was performed using blinded calibrated radiographs by two different observers twice within a period of 2 weeks. The interobserver variability and intraobserver variability (*k* values) were 0.79 and 0.81, respectively. The radiographic evaluation included comparison of preoperative and postoperative scapholunate (SL) gap, scapholunate (SL) angle, lunotriquetral (LT) gap, and carpal height ratio. The SL and LT gaps were measured in the mid-distance of the flat medial facet of the scaphoid and lunate, accordingly [13]. The SL angle was measured by connecting a line tangential to the proximal and distal convexities of the palmar aspect of the scaphoid and another line perpendicular to a line connecting the palmar and dorsal tips of the lunate [14]. The carpal height ratio was calculated by measuring the carpal height and dividing it by the height of the capitate [15].

Cases with radiographically evident scaphoid nonunions, midcarpal, and radiocarpal arthritis were also recorded.

 Statistical analysis was performed using a paired *t* test, with the level of significance set at *P* = 0.05 (SPSS software, version 16.0; SPSS, Chicago, IL).

## Results

At the latest follow-up, the evaluation of the wrist range of motion revealed a mean flexion of 52° (range 25°–70°), which was 79 % compared with the contralateral wrist; the mean extension was 54° (range 30°–70°), which was 80 % of the contralateral wrist; the mean ulnar deviation was 31° (range 18°–42°), which was 81 % of the contralateral wrist, and the mean radial deviation was 18° (range 10°–26°), which was 89 % of the contralateral wrist. The mean pronation was 72° (range 50°–90°), and the mean supination was 80° (range 55°–90°). The mean grip strength was 88 % (range 62–94 %) of the contralateral wrist at the final evaluation.

The radiographic measurements were done immediately after surgery and on the latest postoperative posteroanterior and lateral projections of the wrist. Immediately after surgery, the mean SL gap was 1.6 mm (range 1.3–2 mm), the LT gap was 1.7 mm (range 1.3–2.2 mm), the SL angle was 55^°^ (range 48^°^–61^°^), and the carpal height was 0.54 mm (range 0.52–0.54 mm). The mean SL gap was 1.9 mm (range 1.4–2.6 mm), the mean LT gap was 1.8 mm (range 1.3–2.2 mm), the mean SL angle was 58° (range 48°–64°), and the mean carpal height ratio was 0.52 (range 0.49–0.55) at the latest follow-up. When the measurements of the final radiographs were compared with those of the initial postoperative radiographs, the mean scapholunate gap and scapholunate angle had increased (*P* = .038 and *P* < .001, respectively) and the mean carpal height ratio had decreased statistically significantly (*P* = .038). The increase in scapholunate gap during the follow-up period averaged 0.3 mm (range 0–0.9 mm). The mean scapholunate angle had increased by 3° (range 0°–8°), and the mean carpal height ratio had decreased by 0.02 (range 0–0.04). At the final follow-up, 15 patients had a normal scapholunate angle (normal range 30°–60°) [16] and 16 patients had a normal carpal height ratio (normal range 0.51–0.57) [17]. All radiographic measurements are presented in Table 2.

**Table 2:** Results of radiographic measurements after external fixation and percutaneous K-wire fixation

Parameter	Postoperative mean (range)	Follow-up mean (range)	*P* value
Scapholunate gap	1.6 mm (1.3–2 mm)	1.9 mm (1.4–2.6 mm)	.038
Lunotriquetral gap	1.7 mm (1.3–2.2 mm)	1.8 mm (1.3–2.2 mm)	.295
Scapholunate angle	55o (48o–61o)	58o (48o–64o)	< .001
Carpal height	0.54 (0.52–0.54)	0.52 (0.49–0.54)	.038

Two patients developed radiographically evident arthritis (one patient radiocarpal and one patient midcarpal) at a mean 34 months postoperatively, but it has not been correlated with patients’ pain, range of motion, and wrist function.

The functional outcomes were assessed according to Cooney wrist score [12]. Four patients reported an excellent outcome, eight patients good, and six patients fair, while in two patients, the functional outcome was poor.

Five of seven scaphoid fractures showed radiographically evident union at a mean of 14 weeks (range 9–21 weeks); the other two scaphoid fractures, which were severely comminuted, failed to unite; the first one was a fracture dislocation that was treated with closed reduction, while the second one had an open reduction and internal fixation using a cannulated screw (Acutrak, Acumed Inc., Beaverton, OR, USA) as an initial treatment. Clinically, these two cases showed fair and poor results, respectively. The first one required a salvage operation because of carpal collapse, which consisted of scaphoid excision and midcarpal fusion. The second case showed stable features of a nonunion site with without clinically evident instability of the wrist. Although a surgical option was offered, the patient denied proceeding to surgery. In two patients, skin irritation near the buried pins was developed, which resolved after the pins were removed. Eighteen patients were able to return to their work duties at a mean of 11 weeks (range 8–16 weeks) postoperatively.

## Discussion

Perilunate dislocations and fracture dislocations are injuries related to high-energy trauma of the wrist and may lead to significant functional impairment due to the development of carpal instability and radiocarpal and midcarpal arthritis. Early anatomic reduction of the carpal bones, open repair of interosseous and capsular ligaments, and internal fixation of the fracture has been accepted as a standard surgical method for the treatment of a perilunate dislocation or fracture dislocation [1, 3, 7–9, 18, 19].

Treatment with closed reduction and cast has shown an unacceptable failure rate in terms of quality of reduction and outcome [8]. Minimally invasive procedures have been used in order to minimize surgical trauma and subsequent development of joint stiffness. Arthroscopic reduction of these injuries is emerging [20]. Classical arthroscopic techniques for scapholunate instability consist of debridement, thermal shrinkage, and percutaneous pinning. Good results are obtained in acute lesions or in chronic partial tears. Arthroscopic ligamentoplasty combines the advantages of arthroscopic techniques (minimally invasive surgery) and open techniques (reconstruction of the ligament). With this approach, it is possible to reconstruct the dorsal scapholunate ligament and the secondary stabilizers while causing minimal damage to the soft tissues and avoiding injury to the posterior interosseous nerve and detachment of the dorsal intercarpal ligament. However, this is a technically demanding procedure [21–26].

As an alternative minimally invasive method, we attempted to reduce and fix acute perilunate dislocations or fracture dislocations using a wrist external fixator and percutaneous K-wires fixation. External fixator has been used in difficult fracture dislocations such as: injuries to the “greater arc” with fracture of two or more carpal bones, complete irreducible palmar dislocations associated with intraarticular fractures of the distal radius, and open carpal injuries [12, 27]. It can be also used in polytrauma patients, in associated ipsilateral extremity fractures, when the swelling cause concern about placing the wrist in a cast, in old neglected unreduced trans-scaphoid perilunate dislocations, as well as in compression fracture of the lunate [27, 28]. External fixation facilitates indirect reduction, via ligamentotaxis provided by soft tissue tension to align and hold the reduction and exposure of dislocated carpal bones. The frame is used during the operation as a distraction device to allow indirect reduction. However, after completion of bone and ligament reconstruction, excessive distraction of the intercarpal joints is reduced to minimum level to avoid the development of postoperative stiffness. Furthermore biomechanically, as an adjunctive tool, it proves beneficial because it offers continuous but gentle distraction during the healing process, unloads the damaged carpus and blocks the axial load of the cartilage on the injured proximal carpal row, facilitates the formation of fibrocartilage, enhances joint remodeling, and promotes articular preservation [27, 28].

In our series, external fixator reduced and maintained carpal alignment in all acute perilunate dislocations and in five out of seven trans-scaphoid fracture dislocations. In the other two trans-scaphoid perilunate fracture dislocation, and in one case of trans-styloid perilunate fracture dislocation, an open reduction (through a dorsal approach for scaphoid fractures and volar approach for trans-styloid fracture dislocations) was required. In all 20 wrists, external fixation was augmented with K-wires fixation of intercarpal intervals and for scaphoid and radial styloid fractures.

For the assessment of the effectiveness of this method, we compared radiographic parameters measured on initial postoperative radiographs with those of final radiographs. Although the results showed a tendency toward a loss of reduction with time, this would not show loss of stability because a small change in parameters was observed and the final radiologic values, the final scapholunate gap ,and scapholunate angle, which are the parameters for assessing the scapholunate instability, were also within normal ranges in most patients. Moreover, the mean flexion–extension arc and grip strength in our series were both about 80 and 88 % of the corresponding values for the contralateral wrists. Carpal alignment could be effectively restored with an external fixator, and reduction was maintained within normal ranges in 20 patients at a mean follow-up of 39 months. The clinical results of our series compare favorably with those of open and arthroscopically assisted techniques [10, 29, 30].

Many surgeons have emphasized the need for repair of the interosseous ligament in order to stabilize the carpal architecture [6, 19], whereas some investigators suggested that maintenance of the anatomic position is sufficient to re-establish carpal stability without individual ligament repair [3, 8]. The results of our study suggest that the capsular structures can be healed adequately with a good vascularity when they are accurately approximated and protected for some period and open repair of interosseous ligament is not necessary. In this study, all the patients were treated within 8 h (range 2–12 h) from the injury. The great advantage of the method is that it facilitates the union of fractures and intercarpal ligaments because it can lessen capsular and adjacent soft tissue injury and provide preservation of an already tenuous blood supply.

Although functional outcomes are not well related with radiologic results, post-traumatic arthritis has been reported to be common after these injuries, with rate of radiographic arthritis from 50 to 86 % after open reduction and internal fixation [4, 9, 10]. In our study, two of 20 injured wrists had radiographic evidence of midcarpal and radiocarpal arthritis at a mean follow-up of 39 months, with no clinical relevance.

The retrospective nature and the different types of perilunate dislocations included into our cohort consist the main limitations of our study. However, we believe that this is a concise presentation of a relatively large sample of these uncommon and severe carpal injuries that were managed following the same basic concept of treatment: the use of external fixation, minimal soft tissue detachments, and sufficient follow-up to assess for early complications.

## Conclusion

In acute perilunate dislocations and fracture dislocations, carpal alignment could be effectively restored and maintained using a wrist external fixator augmented with K-wires. Indirect reduction via ligamentotaxis probably enhances intercarpal ligaments healing, without the need for an open repair, thus avoiding additional soft tissue detachments and soft tissue damage.
